# Laxative and antioxidant effects of ramie (*Boehmeria nivea* L*.*) leaf extract in experimental constipated rats

**DOI:** 10.1002/fsn3.1619

**Published:** 2020-05-07

**Authors:** Hyun‐Joo Lee, Eun Joo Choi, Sihoon Park, Jae‐Joon Lee

**Affiliations:** ^1^ Department of Nutrition and Culinary Science Hankyong National University Ansung Korea; ^2^ Department of Pharmacy College of Pharmacy Chosun University Gwangju Korea; ^3^ Department of Food and Nutrition Chosun University Gwangju Korea

**Keywords:** constipation, loperamide, oxidative stress, ramie leaf extract, rat

## Abstract

Ramie leaf (*Boehmeria nivea* L.) is rich in cellulose, polyphenol compounds, vitamin C, and minerals. The leaves of this plant, which are used for medicinal purposes, have long been reported to have anti‐inflammatory, antioxidant, anticolitis, and antidiabetic effects. We investigated the protective effects of ramie leaf ethanol extract (RLE) against loperamide‐induced constipation and oxidative stress in rats. Male Sprague‐Dawley rats were administered 200 or 400 mg/kg body weight of RLE (RLEL and RLEH groups) by gavage, while normal (NOR) and control (CON) rats received saline. Loperamide (4.0 mg/kg, twice per day) was injected subcutaneously to induce constipation in RLEL, RLEH, and CON groups. Total fecal number, wet weight, and water content decreased, while the total number of loperamide‐induced fecal pellets in the distal colon increased with administration of RLE in a dose‐dependent manner. Gastrointestinal transit time was more greatly reduced in RLE‐treated groups than in the CON group. Serum total cholesterol (TC) level, as well as alanine aminotransferase (ALT) and alkaline phosphatase (ALP) activity, was significantly lower in both RLEL and RLEH groups compared with the CON group. Intestinal mucosa malondialdehyde (MDA) and hydrogen peroxide (H_2_O_2_) production decreased significantly in a dose‐dependent manner in the RLE‐treated groups. Loperamide decreased the antioxidant enzyme activity, including that of superoxide dismutase (SOD) and glutathione peroxidase (GSH‐Px), while RLE administration increased the antioxidant activity. These results suggest that RLE exerts potent laxative and antioxidant effects in model rats with loperamide‐induced constipation.

## Graphical abstract

Oral treat of RLE increased the total number, weight, and water content of rat fecal pellets without causing diarrhea, the incidence of which was decreased by loperamide. Treatment with RLE significantly protected against oxidative stress induced by loperamide intoxication. Phenolic compounds and dietary fiber in RLE may play key roles in the observed anticonstipation and antioxidative effects.

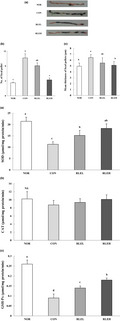

## 1 INTRODUCTION

In Korea, the prevalence of various diseases, including gastrointestinal disorders and constipation, has increased due to increased work‐related stress and the growing popularity of a westernized diet. Constipation is often accompanied by a lack of appetite and persistent abdominal bloating. Severe constipation may cause anal fissures or prolapsed hemorrhoids during defecation, and in extreme cases can lead to colorectal cancer (Corazziari, 1999). Defined as two or fewer bowel movements per week with a stool weight of <35 g, constipation often occurs due to a lack of exercise or accumulated stress, leading to decreased gastrointestinal movement and impaired ability to defecate (Corfield, Carroll, Myerscouh, & Probert, 2001). As laxative drugs may cause severe side effects, medicinal foods and plants with laxative effects are worth investigating as alternative treatments for constipation.

The herb *Boehmeria nivea*, also called ramie, is a perennial dicotyledon of the Urticaceae family (Wei et al., 2014). Ramie originates from South‐East Asia, is distributed in Korea, China, the Philippines, and India, and inhabits warm and humid regions (Zhao et al., 2008). In Korea, ramie leaf has been used to make tea, rice cakes, and clothes. Ramie leaves, used for medicinal and edible purposes, are effective in reducing fever, hematemesis, extravasation, constipation, diarrhea, and toxicity neutralization (Cho et al., 2017;Lee, Lee, Cho, Kim, & Choi, 2018; Lee et al., 2009; Lee, Woo, Jeong, & Kim, 2010). Furthermore, the plant is used to treat hemoptysis, bleeding, small abscesses, hematuria, anal edema, pain, and bruising (Lin, Yen, Lo, & Lin, 1998). Recently, ramie leaf was reported to exert antioxidant (Chen et al., 2014; Lin et al., 1998; Wang et al., 2015, 2019), anti‐inflammatory (Sung, Davaatseren, Kim, Kim, & Hwang, 2013), antihepatitis B virus (Wei et al., 2014), anticolitis (Shin, Sung, Yang, Kim, & Hwang, 2014), antiobesity (Lee, Kim, & Lee, 2016), neuroprotective (Wang et al., 2015), and antidiabetic effects (Kim, Lee, et al., 2013;Kim, Sung, Park, Yang, & Hwang, 2013; Lee, Kim, & Lee, 2014). Ramie leaf is rich in dietary fiber, vitamin C, and phytochemicals, including benzoic acid, 4‐coumaric acid, caffeic acid, ferulic acid, rutin, chlorogenic acid, catechin, epicatechin, epicatechin gallate, and β‐sitosterol (Chen et al., 2014; Lee et al., 2009, 2015;Wang et al., 2019). Mohanty, Misra, and Hinrichsen (2000) showed that ramie leaf contains several dietary fibers, such as celluloses (68.6%–76.2%), hemicelluloses (13.1%–16.7%), pectin (1.9%), wax (0.3%), and lignin (0.6%–0.7%).

Certain bioactive compounds have been reported to exert both laxative and antioxidative properties. These compounds include the following: flavonol naringenin extracted from citrus fruits (Yang et al., 2008); mangiferin and genkwanin 5‐*O*‐β‐primeveroside extracted from agarwood leaf (Ito et al., 2012; Kakino et al., 2010); flavonoids, terpenoids, tannins, phenols, gum, and mucilage extracted from *Ageratum conyzoides* L. (Sathyanathan, Satish, Eswar Kumar, Shuchrajit, & Thilothama, 2013); and polyphenols, polydatin, and resveratrol extracted from peanut sprout (Seo et al., 2013). Furthermore, Attaluri, Donahoe, Valestin, Brown, and Rao (2011) reported that large amounts of dietary fiber, fructan sorbitol, and phenolic compounds (184 mg/100 g), particularly neochlorogenic acid and chlorogenic acid, may assist in producing laxative effects.

Oxidative stress leads to intestinal dysmotility and has been observed in animals with constipation as well as in those with colorectal cancer and other chronic illness associated with constipation (Li, Zong, Qi, & Liu, 2011). Intestinal oxidative stress has been induced under experimental circumstances using several agents: castor oil, acetic acid, ethanol, aspirin, and loperamide (Jabri et al., 2017).

It is necessary to study plant‐based natural products and foods that are effective in preventing and treating gastrointestinal disorders. Based on our results, we hypothesized that RLE could be a potential candidate for alleviation of constipation. Therefore, the present study was conducted to investigate the laxative and antioxidant effects of RLE on loperamide‐induced constipation in rats.

## 2 MATERIALS AND METHODS

### 2.1 Preparation of ramie leaf extracts

Ramie leaf (*Boehmeria nivea* L.) was purchased from the Nokdang Bio Foods Farming Association Corporation. Leaves were washed twice, initially dried using a salad spinner (Windax, Roichen Co.), further dried using a lyophilizer (ED 8512, Ilshin Co.), triturated using a grinder (HR2904, Royal Philips Electronics NV), freeze‐dried, and then pulverized into a powder. Powdered ramie leaves (100 g) were extracted with 1,500 ml of 80% ethanol solution with agitation at 60°C for 3 hr. Extracts were then filtered, concentrated using a rotary evaporator (EYELA Vacuun Controller NVC‐1100, EYELA), freeze‐dried, and stored at −70°C prior to use as a sample.

### 2.2 Determination of total polyphenol, total flavonoid, chlorogenic acid, and dietary fiber contents

Total polyphenol content was determined using Folin–Ciocalteu's reagent (Folin & Denis, 1912), and total flavonoid content was measured in accordance with the Davis method (Chae et al., 2002), with slight modifications. Total polyphenol and flavonoid contents are expressed as mg tannic acid equivalent (TAE) and mg rutin equivalent (RE) per gram of sample, respectively. Total chlorogenic acid content was measured in accordance with the Coseteng method (Coseteng & Lee, 1987), with slight modifications, using chlorogenic acid as an external standard. Dietary fiber was determined using a slightly modified AOAC enzymatic‐gravimetric method (Prosky et al., 1985).

### 2.3 Animals and induction of constipation

Male Sprague‐Dawley (*SD*) rats (*n* = 32), with an average weight of 170 g, were purchased from Central Laboratory Animal Inc. (SLC Inc.). Rats were provided with a standard formula feed and allowed a 1‐week adaptation period to the environment. Rats were selected on body weight basis (mean; 213.01 ± 8.23 g), separated from each other into single‐occupancy stainless steel metabolic cages, and fed a commercial pellet diet for 34 days. Rats were divided into four groups (*n* = 8 per group): The NOR group was fed a normal diet without induction of constipation for the duration of the experiment, whereas the CON group was fed a normal diet and constipation was induced with loperamide (Table 1). RLE was orally administered at 200 or 400 mg kg^−1^ day^−1^ (RLEL (low) and RLEH (high) groups, respectively) for 34 days. RLE was suspended in distilled water at concentration of 200 or 400 mg/kg body weight/day and administered at a volume of 1.0 ml/200 g body weight of rat. Rats were fed for 28 days without loperamide, and then, constipation was induced in the CON, RLEL, and RLEH groups. Over a period of 6 days, starting on day 29, loperamide dissolved in 0.9% saline was subcutaneously injected at 4.0 mg/kg of body weight twice per day (at 9:00 a.m. and 6:00 p.m.). The NOR group was injected with 0.9% saline without loperamide in the same manner as the experimental rats. Conditions of the experimental animal breeding room were maintained at 22.0 ± 1°C, 65.0 ± 5% relative humidity, with a 12 hr‐light cycle (light from 09:00 to 21:00 hr). Food pellets (Samyang Foods Co. Ltd.) and tap water were supplied ad libitum during the experimental period. Body weight, food intake, water intake, and fecal biomarkers for each rat were measured once per week before constipation induction, but daily after loperamide‐induced constipation.

**TABLE 1:** Experimental design

Groups	Diet composition
NOR	Normal diet
CON	Normal diet and loperamide‐treated group
RLEL	Ramie leaf extract 200 mg kg^−1^ day^−1^ and loperamide‐treated group
RLEH	Ramie leaf extract 400 mg kg^−1^ day^−1^ and loperamide‐treated group

### 2.4 Blood sample and tissue collection

At the end of experiments, animals were fasted for 12 hr, blood samples were collected, and serum was processed for lipid profile determination. Distal portions of the colon were removed and cut longitudinally, cleaned with saline solution to remove fecal residues, and homogenized on ice with phosphate‐buffered saline (PBS, pH 7.4) to obtain 1:9 (w/v) whole homogenate that was then centrifuged at 10,000 g for 15 min at 4°C. The supernatants were used for biochemical determination of malondialdehyde (MDA) and hydrogen peroxide (H_2_O_2_) levels, as well as superoxide dismutase (SOD), catalase (CAT), and glutathione peroxidase (GSH‐Px) activity.

### 2.5 Measurement of fecal parameters

To collect pure feces without any contamination, all rats were housed in individual metabolic cages (Daejong Instrument Industry Co., Ltd.). The total number, wet weight, and water content of the excreted fecal pellets were measured in two phases: before and after the induction of constipation. Fecal pellets from each rat were collected from the first day of the experiment, four times per day at intervals, every day during the constipation induction. The number and weight of the collected fecal pellets were measured. To measure the stool water content, fecal pellets were dried at 70°C for 24 hr in an oven and then weighed. Water content of the pellets was then determined by subtracting the mass of the dried feces from that of the wet feces (Rtibi et al., 2017).

### 2.6 Number and thickness of intestinal fecal pellets

The total number and mean thickness (short axis) of fecal pellets in the large intestine were determined by excising the entire intestine from the appendix to the rectum on the sixth day after the loperamide administration. The intestinal tissue was then fixed in a solution of 10% formaldehyde in phosphate‐buffered saline (PBS, pH 7.4) to preserve the total number of fecal pellets in the colon (Hinton, Lennard‐Jones, & Young, 1969).

### 2.7 Measurement of intestinal transit time and length

The gastrointestinal transit time of feed consumed by the rats was measured using the method of Hinton et al. (1969), with minor modification. The animals were fasted for 12 hr on the sixth day after administration of loperamide. Rats were then fed a diet mixed with 1 g of 10% Coomassie brilliant blue dye, and the total time taken to defecate the blue‐colored fecal pellets was determined to be the gastrointestinal transit time. Intestinal length was measured by resection (excluding the appendix).

### 2.8 Measurement of serum biochemical parameters

The serum triglyceride (TG), total cholesterol (TC), and high‐density lipoprotein (HDL)‐cholesterol contents, as well as alanine aminotransferase (ALT), aspartate aminotransferase (AST), alkaline phosphatase (ALP), and lactate dehydrogenase (LDH) activities, were measured using a blood biochemistry automatic analyzer (Fuji Dri‐Chem 3500s, Fujifilm).

### 2.9 Thiobarbituric acid‐reactive substance (TBARS) and hydrogen peroxide production assay

Malondialdehyde (MDA) concentration was measured in samples of intestinal mucosa according to the method described by Draper and Hardly (1990). The reaction with thiobarbituric acid‐reactive substance (TBARS) was measured in terms of absorbance at 532 nm using a UV‐visible spectrophotometer (DU‐650, Beckman). Results were expressed as mean of MDA nmol/mg of protein. Hydrogen peroxide (H_2_O_2_) content was assessed according to Dingeon, Ferry, and Roullet (1975), and results were expressed as μmol of H_2_O_2_ per mg of protein. Protein content was assayed by the method of Hartree (1972), which is a slight modification of the Lowry method.

### 2.10 Oxidative stress marker assessment

Superoxide dismutase (SOD) activity in colon homogenates was assessed based on the colorimetric reaction inhibition of adrenaline according to the method of Misra and Fridovich (1972), and CAT activity in colon homogenates was analyzed by monitoring the decomposition rate of hydrogen peroxide according to the modified method of Beers and Sizer (1952). Glutathione peroxidase (GSH‐Px) activity was examined using the procedure of Flohé and Günzler (1984). SOD, CAT, and GSH‐Px activities are expressed as units per mg of protein.

### 2.11 Statistical analysis

All data were subjected to general linear model (GLM) analysis computed using the Statistical Analysis System (SAS) package and are presented as mean ± *SE*. One‐way analysis of variance was conducted to evaluate differences in means among three or more groups, and significance of the intergroup mean differences was tested using Tukey's test at *p* < .05.

## 3 RESULTS

### 3.1 Total polyphenol, total flavonoid, chlorogenic acid, and dietary fiber content of RLE

The total polyphenol, flavonoid, and chlorogenic acid contents in RLE were found to be 141.43 ± 1.96 mg of TAE/g dry matter (DM), 106.38 ± 2.01 mg of RE/g DM, and 19.65 ± 0.41 mg/g DM, respectively (Table 2). The dietary fiber content of RLE was 12.51%, composed of insoluble (7.67%) and soluble (4.84%) dietary fibers.

**TABLE 2:** Content of total polyphenols, total flavonoids, chlorogenic acid, and dietary fiber in ramie leaf ethanol extract

Sample	Total polyphenol (mg of TAE/g DM)	Total flavonoid (mg of RE/g DM)	Chlorogenic acid (mg/g DM)	Dietary fiber (%, DM)		
				Total dietary fiber^1^ (TDF)	Insoluble dietary fiber (IDF)	Soluble dietary fiber (SDF)
RLE	141.43 ± 1.96^2^	106.38 ± 2.01	19.65 ± 0.41	12.51 ± 2.01	7.67 ± 1.03	4.84 ± 1.33

^1^ Calculated using the following equation: TDF = IDF + SDF.

^2^ All values are expressed as mean ± *SE* of triplicate measurements.

### 3.2 Effect of RLE on body weight gain, food intake, and water intake

Body weight gain, food intake, and water intake of *SD* rats administered RLE before and after injection of loperamide are shown in Table 3. Before loperamide‐induced constipation, body weight gain decreased more in the RLEL and RLEH groups than in the NOR and CON groups, which received a normal diet. After the induction of loperamide‐induced constipation, the body weight gain significantly decreased in the CON group compared with the NOR, RLEL, and RLEH groups. Body weight gains increased significantly in the NOR, RLEL, and RLEH groups by 58.0%, 42.2%, and 43.6%, respectively, compared with the CON group. Before the induction of constipation, no significant changes in food intake or water consumption occurred between the RLE‐treated groups and the NOR and CON groups. After the induction of constipation, food intake decreased more significantly in the CON group than in the NOR, RLEL, and RLEH groups. Water consumption decreased by 13.5% in the CON group compared with the NOR group. RLE administration prevented decreased water consumption in a dose‐dependent manner.

**TABLE 3:** Effects of ramie leaf extract on body weight gain, food intake, and water consumption in rats

	Groups^1^			
	NOR	CON	RLEL	RLEH
Before constipation (days 0–28)				
Initial body weight (g)	213.01 ± 8.38	212.29 ± 9.78	212.85 ± 7.65	213.93 ± 9.47
Final body weight (g)	380.2529 ± 10.29^2^ ^a^ ^3^	378.33 ± 12.01^a^	365.77 ± 14.33^ab^	357.57 ± 11.28^b^
Body weight gain (g/day)	5.93 ± 0.44^a^	5.97 ± 0.18^a^	5.46 ± 0.35^ab^	5.13 ± 0.15^b^
Food intake (g/day)	25.29 ± 5.87^NS^ ^4^	24.98 ± 2.51	24.42 ± 1.98	24.90 ± 3.82
Water consumption (ml/day)	8.43 ± 1.14 ^NS^	8.26 ± 2.87	8.24 ± 1.66	8.01 ± 1.92
After constipation (days 29–33)				
Body weight gain (g/day)	5.86 ± 0.28^a^	3.71 ± 0.37^c^	4.12 ± 0.24^b^	4.08 ± 0.12^b^
Food intake (g/day)	24.01 ± 3.25^a^	19.28 ± 1.87^b^	20.57 ± 1.66^a^	23.12 ± 2.54^a^
Water consumption (ml/day)	8.35 ± 1.17^a^	7.22 ± 1.52^b^	7.67 ± 1.92^b^	8.21 ± 1.03^a^

^1^ NOR, normal diet group; CON, normal diet and loperamide‐treated group; RLEL, ramie leaf extract 200 mg kg^−1^ day^−1^ and loperamide‐treated group; RLEH, ramie leaf extract 400 mg kg^−1^ day^−1^ and loperamide‐treated group.

^2^ Mean ± *SE* (*n* = 8).

^3^ Values with different superscripts in the same row are significantly different (*p* < .05) between groups as determined by Tukey's test.

^4^ NS: not significantly different among groups.

### 3.3 Effect of RLE on total number, weight, and water content of fecal pellets

**FIGURE 1:**
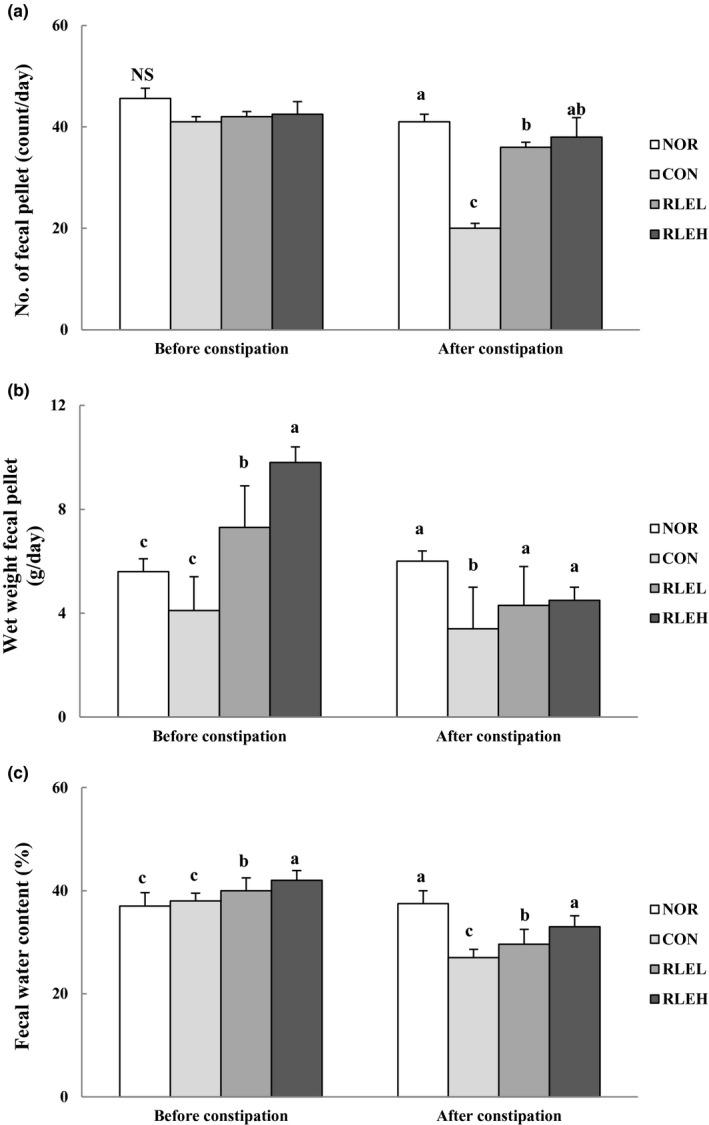
Effects of ramie leaf extract on number of fecal pellets (a), wet weight of fecal pellets (b), and fecal water content (c) in rats. NOR, normal diet group; CON, normal diet and loperamide‐treated group; RLEL, ramie leaf extract 200 mg kg^−1^ day^−1^ and loperamide‐treated group; RLEH, ramie leaf extract 400 mg kg^−1^ day^−1^ and loperamide‐treated group. The values are the mean ± *SE* of eight rats per group, and different superscript letters indicate significant differences at *p* < .05 as determined by Tukey's test

The total number, weight, and water content of fecal pellets before and after loperamide injection in rats administered RLE are shown in Figure 1. Before loperamide injection, the total number of fecal pellets did not significantly differ among the groups. However, fecal weight and water content increased more in RLE‐treated groups than in NOR and CON groups. RLE administration prevented decreased fecal pellet weight and water content in a dose‐dependent manner. After loperamide injection, the total number and weight of fecal pellets tended to increase with increased RLE dose, and fecal water content was higher in the RLEL and RLEH groups than in the CON group. In addition, after induction of constipation, the total number, weight, and water content of fecal pellets in the RLEH group were similar to those in the NOR group, in which constipation was not induced.

### 3.4 Effect of RLE on total number and mean thickness of colonic fecal pellets

**FIGURE 2:**
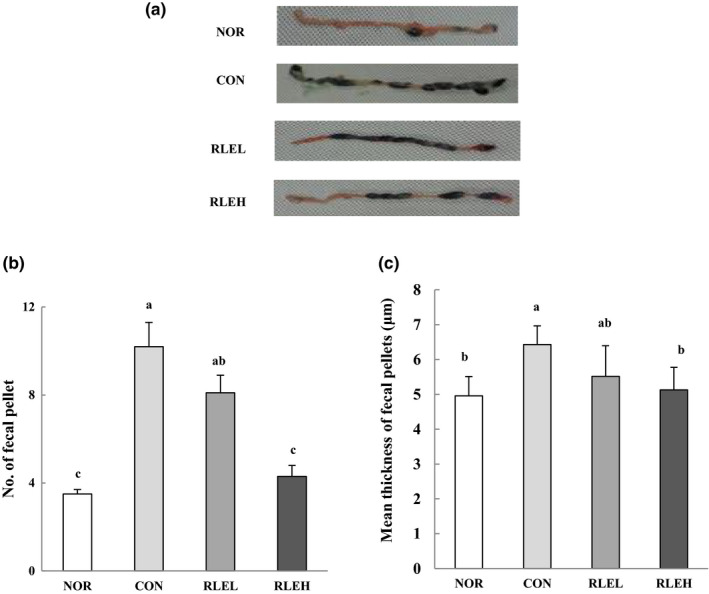
Effects of ramie leaf extract on isolated distal colon with fecal pellets (a), and the number (b), and mean thickness (c) of fecal pellets in loperamide‐induced rat colon for 6 days. NOR, normal diet group; CON, normal diet and loperamide‐treated group; RLEL, ramie leaf extract 200 mg kg^−1^ day^−1^ and loperamide‐treated group; RLEH, ramie leaf extract 400 mg kg^−1^ day^−1^ and loperamide‐treated group. The values are the mean ± *SE* of eight rats per group, and different superscript letters indicate significant differences at *p* < .05 as determined by Tukey's test

The total number and mean thickness of fecal pellets remaining in the colon of RLE‐treated rats with loperamide‐induced constipation are shown in Figure 2. The CON group demonstrated fecal congestion in the colon and defecated fewer times than the NOR group. However, the number of fecal pellets in the colon was more reduced in the RLEL and RLEH groups than in the CON group. In particular, the number of fecal pellets in the RLEH group was similar to that in the NOR group. The mean thickness of fecal pellets remaining in the colon lumen was increased by 29.6% in the CON group compared with the NOR group. However, the mean thickness of fecal pellets was decreased by 14.2% and 20.2% in the RLEL and RLEH groups, respectively, when compared with the CON group.

### 3.5 Effect of RLE on gastrointestinal transit time and intestine length

**FIGURE 3:**
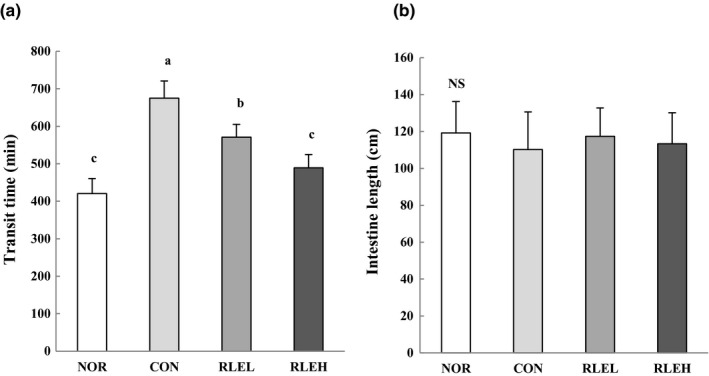
Effects of ramie leaf powder on gastrointestinal transit time (a) and intestine length (b) in loperamide‐induced constipation in rats. NOR, normal diet group; CON, normal diet and loperamide‐treated group; RLEL, ramie leaf extract 200 mg kg^−1^ day^−1^ and loperamide‐treated group; RLEH, ramie leaf extract 400 mg kg^−1^ day^−1^ and loperamide‐treated group. The values are the mean ± *SE* of eight rats per group, and different superscript letters indicate significant differences at *p* < .05 as determined by Tukey's test. NS: not significantly different among groups

Gastrointestinal transit time and intestinal length of rats are shown in Figure 3. Gastrointestinal transit time was more greatly reduced in RLE‐treated groups than in the CON group, and this difference was significant. In addition, the gastrointestinal transit time in the RLEH group was similar to that in the NOR group. However, intestinal length was not significantly changed among experimental groups.

### 3.6 Effect of RLE on serum biochemical parameters

The effects of RLE on serum lipid profiles and cardiac biomarkers are presented in Table 4. Serum TG levels were not significantly different among experimental groups. Serum TC levels were generally lower in RLE‐treated groups than in the NOR and CON groups. Serum HDL cholesterol level significantly increased in the NOR, RLEL, and RLEH groups compared with the CON group. In addition, AI and CRF values were significantly higher in CON group than in RLE‐treated groups. Serum ALT and ALP activities decreased in the RLEL and RLEH groups compared with that in the CON group. Serum AST and LDH activities were not significantly changed among experimental groups.

**TABLE 4:** Effect of ramie leaf extract on serum lipid profiles

	Groups^1^			
	NOR	CON	RLEL	RLEH
Serum lipid profiles (mg/dL)				
Triglyceride	90.12 ± 12.82^4^ ^a^ ^5^	97.36 ± 9.89^a^	87.01 ± 10.21^ab^	90.45 ± 8.44^b^
Total cholesterol	92.97 ± 8.21^a^	102.08 ± 9.78^a^	85.13 ± 6.87^b^	81.27 ± 8.27^b^
HDL cholesterol	43.26 ± 6.01^a^	35.90 ± 5.12^b^	42.34 ± 4.29^a^	45.47 ± 5.98^a^
AI^2^	1.26 ± 0.27 ^ab^	1.86 ± 0.59^a^	1.01 ± 0.30^b^	0.97 ± 0.08^b^
CRF^3^	2.15 ± 0.77^b^	2.84 ± 0.59^a^	2.01 ± 0.11^b^	1.78 ± 0.10^c^
Serum cardiac biomarkers (U/L)				
ALT	39.65 ± 3.28^a^	43.26 ± 4.98^a^	32.18 ± 2.17^b^	30.33 ± 2.99^b^
AST	49.23 ± 4.11^NS^ ^6^	52.21 ± 5.87	48.29 ± 4.12	47.22 ± 3.21
ALP	501.23 ± 15.33^b^	592.32 ± 20.13^a^	499.23 ± 18.47^b^	452.32 ± 18.21^c^
LDH	704.23 ± 35.69^NS^	724.63 ± 29.78	702.36 ± 28.46	695.23 ± 30.22

^1^ NOR, normal diet group; CON, normal diet and loperamide‐treated group; RLEL, ramie leaf extract 200 mg kg^−1^ day^−1^ and loperamide‐treated group; RLEH, ramie leaf extract 400 mg kg^−1^ day^−1^ and loperamide‐treated group.

^2^ AI (Atherogenic index) = (Total cholesterol − HDL cholesterol)/HDL cholesterol.

^3^ CRF (cardiac risk factor) = Total cholesterol/HDL cholesterol.

^4^ Mean ± *SE* (*n* = 8).

^5^ Values with different superscripts in the same row are significantly different (*p* < .05) between groups as determined by Tukey's test.

^6^ NS: not significantly different among groups.

### 3.7 Effect of RLE on colic lipid peroxidation and H_2_O_2_ production

**FIGURE 4:**
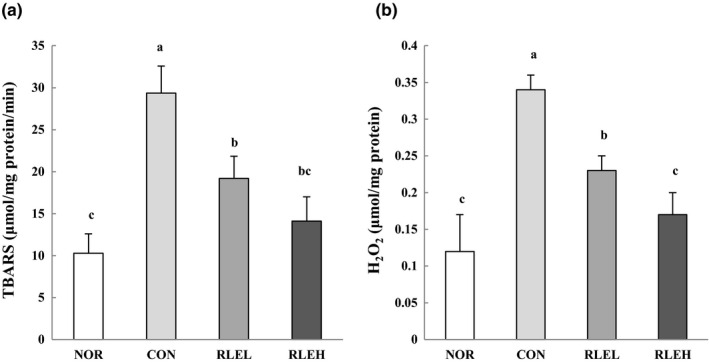
Effects of ramie leaf extract on intestinal mucosa MDA (a) and hydrogen peroxide (b) levels during loperamide‐induced constipation. NOR, normal diet group; CON, normal diet and loperamide‐treated group; RLEL, ramie leaf extract 200 mg kg^−1^ day^−1^ and loperamide‐treated group; RLEH, ramie leaf extract 400 mg kg^−1^ day^−1^ and loperamide‐treated group. The values are the mean ± *SE* of eight rats per group, and different superscript letters indicate significant differences at *p* < .05 as determined by Tukey's test

The lipid peroxidation (TBARS) concentration in the CON group was 29.36 μm/mg protein/min in the intestinal mucosa. However, this same parameter was 10.29 μm/mg protein/min in the intestinal mucosa in the NOR group (Figure 4). When RLE concentration was increased from 200 to 400 mg/kg, production of lipid peroxide was decreased from 19.21 to 14.11 μm/mg protein/min in the intestinal mucosa, a decrease of ~34.5%–51.9% compared with the CON group. Increased H_2_O_2_ was produced in CON and RLEL groups after loperamide intoxication compared with the NOR group. RLEL and RLEH groups demonstrated significant protection by reducing the elevated content of H_2_O_2_ when compared with the CON group.

### 3.8 Effect of RLE on antioxidant enzyme activities

**FIGURE 5:**
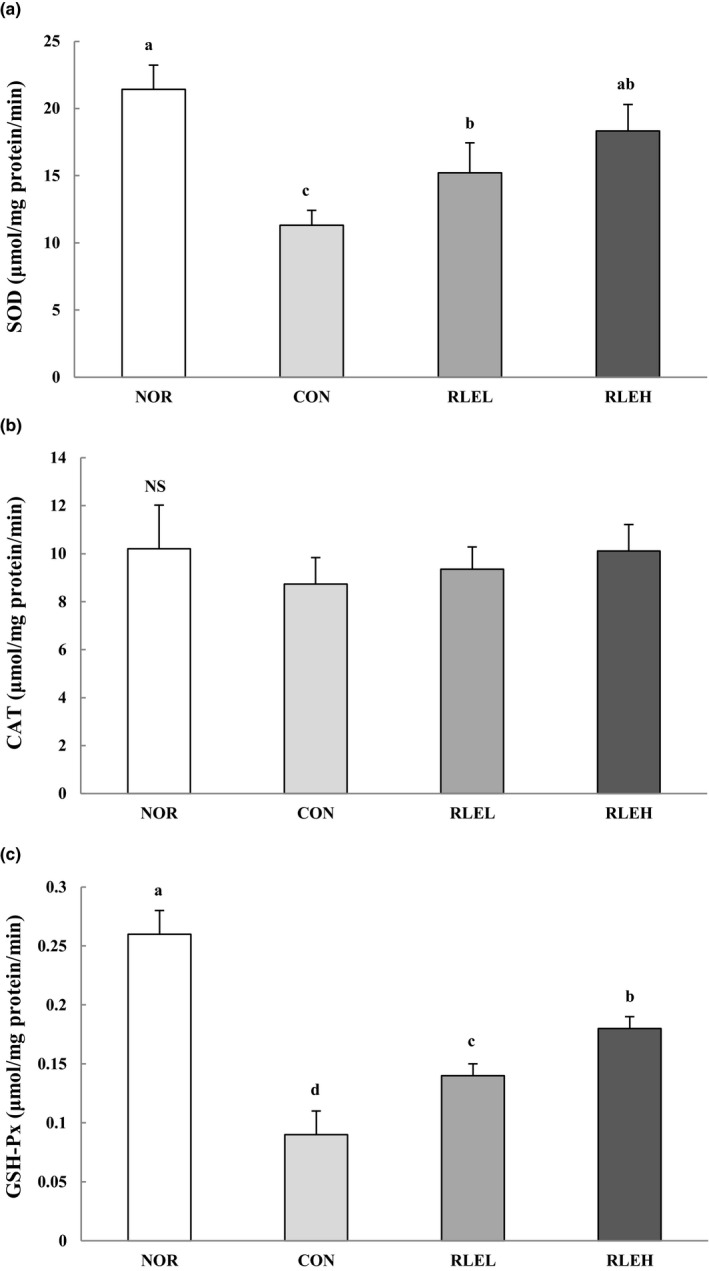
Effects of ramie leaf extract on intestinal mucosa SOD (a), CAT (b), and GSH‐Px (c) activity during loperamide‐induced constipation. NOR, normal diet group; CON, normal diet and loperamide‐treated group; RLEL, ramie leaf extract 200 mg kg^−1^ day^−1^ and loperamide‐treated group; RLEH, ramie leaf extract 400 mg kg^−1^ day^−1^ and loperamide‐treated group. The values are the mean ± *SE* of eight rats per group, and different superscript letters indicate significant differences at *p* < .05 as determined by Tukey's test

Changes in enzymatic antioxidant activities in the intestinal mucosa of the experimental groups are shown in Figure 5. SOD activity in the CON group was 11.32 units/min/mg protein, a decrease of ~47.1% compared with the NOR group. On the other hand, when the RLEL and RLEH groups were administered RLE, SOD activities were 15.22 and 18.33 units/min/mg protein, showing an increase of ~34.5% and ~61.9%, respectively, compared with the CON group. The activity of CAT was not significantly different among the experimental groups. The activity of GSH‐Px in the CON group was significantly reduced when compared with the NOR group. However, the activity of this enzyme increased in a significant and dose‐dependent manner in groups treated with RLE.

## 4 DISCUSSION

Various medicinal foods and plants containing many bioactive compounds have recently emerged as novel therapeutic materials for the treatment or prevention of constipation (Aslam & Janbaz, 2019; Attaluri et al., 2011; Kakino et al., 2010; Kim, Lee, et al., 2013; Kim, Sung, et al., 2013; Langmead & Rampton, 2001; Méité et al., 2010; Sathyanathan et al., 2013; Seo et al., 2013). Polyphenol‐rich dietary sources include such materials as agarwood (Kakino et al., 2010), *Aloe ferox* Mill. (Wintola, Sunmonu, & Afolayan, 2010), *Ageratum conyzoides* L. (Sathyanathan et al., 2013), peanut sprout (Seo et al., 2013), *Liriope platyphylla* [33], *Mareya micrantha* (Benth.) müll. Arg. (Euphorbiaceae) (Méité et al., 2010), *Malva sylvestris* (2017), and cactus (*Opuntia humifusa*) (Han et al., 2017). These are known to exhibit laxative properties based on their ability to increase fecal volume, intestinal motility, frequency of feces, and ileal tension. Many studies have attributed the laxative properties of plants to the presence of naringenin, mangiferin, genkwanin‐5‐*O*‐β‐primeveroside, flavonoids, terpenoids, tannin, phenols, gum, mucilage, polydatin, resveratrol, fructan, sorbitol, neochlorogenic acid, quercetin, and chlorogenic acid (Kakino et al., 2010; Kim et al., 2018; Sathyanathan et al., 2013; Seo et al., 2013). RLE has been reported to contain high levels of polyphenolic and flavonoid compounds (Chen et al., 2014;Lee et al., 2009, 2014, 2015; Sung et al., 2013; Tan et al., 2014). The leaves of this plant, traditionally used for medicinal purposes, have long been reported to exert laxative effects. In this study, RLE was found to contain 141.43 ± 1.96 mg of TAE/g DM total polyphenol, 106.38 ± 2.01 mg of RE/g DM total flavonoid, and 19.65 ± 0.41 mg/g DM chlorogenic acid. In addition, the dietary fiber content of RLE was 12.51%, composed of insoluble (7.67%) and soluble (4.84%) dietary fibers. In this study, we investigated the protective effects of polyphenol compounds and dietary fiber‐rich RLE on loperamide‐induced constipation and oxidative stress in rats.

Rats administered the higher dose of RLE prior constipation induction experienced lower body weight gain than rats in other groups. Consumption of dietary fiber and polyphenol compounds is known to inhibit weight gain (Gabel et al., 1997;Tuzcu, Orhan, Sahin, Juturu, & Sahin, 2017) and to exert antiobesity effects (Blaak & Saris, 1995;Sun, Wu, & Chau, 2016); RLE produced similar effects. After constipation induction, body weight gain, food intake, and water consumption decreased more in the normal diet and loperamide‐treated group (CON) than in the other groups. These findings were similar to those of Jeon, Kim, and Choi (2007), which indicated that loperamide‐induced constipation tended to reduce food intake and subsequently affected body weight gain. These findings were also similar to Wintola et al. (2010). Food intake and water consumption are considered important factors for the assessment of constipation symptoms and therapeutic effects (Kim, Lee, et al., 2013; Kim, Sung, et al., 2013). In our study, food intake and water consumption increased in response to RLE treatment after constipation induction.

Our results demonstrated that RLE exerts laxative effects without deleterious side effects. We found that administering loperamide to induce constipation generally reduced the total number, weight, and water content of fecal pellets. A previous study reported that loperamide likely increases colonic water absorption (Theoduro, Fioramonti, Hachet, & Bueno, 1991) or inhibits secretion (Read, 1983), in addition to reducing intestinal motility. In agreement with Shimotoyodome, Meguro, Hase, Tokimitsu, and Sakata (2000), decreased fecal excretion was thought to result not from decreased food intake, but from inhibition of colonic peristalsis by loperamide. Loperamide is an opioid meperidine derivative with almost no analgesic effect that is used as an antidiarrheal drug. Moreover, loperamide inhibits peristalsis through direct action on smooth muscle, causing a delay in the small intestine transit time (Schiller, Santa Ana, Moravski, & Fordtran, 1984). Therefore, loperamide is useful in creating an experimental model that involves inhibition of motor function of the intestine.

Symptoms associated with constipation were alleviated by treatment with RLE. After the induction of constipation, the total number, weight, and water content of the fecal pellets obtained from the RLEH group were similar to those of the NOR group. This result was similar to that reported by Yook et al. (2003), which indicated that rats fed a diet containing a large amount of dietary fiber showed a high fecal pellet weight, as well as a relatively high fecal pellet water content. Previously, the number of fecal pellets remaining in the colon, a key biomarker of constipation, was markedly increased in rats after administration of loperamide (Lee et al., 2012). However, this result was significantly reduced by RLE treatment. The RLE groups showed a dose‐dependent reduction in the number of fecal pellets in the colon. In addition to dietary fiber, antioxidant properties may also contribute to the effects of RLE against constipation. Numerous studies of gastrointestinal function have shown that polyphenols have potential health benefits via modulation of the gut microbiota and the existence of prebiotic‐like effects (Tabasco et al., 2011; Tzounis et al., 2011). Peanut sprout extract, which contains a high level of polyphenolic compounds, increased fecal frequency, number, and water content in rats with loperamide‐induced constipation (Seo et al., 2013). Thus, we suggest that polyphenolic compounds, the main bioactive components in RLE, may be responsible for this effect. These results demonstrate that RLE reduces loperamide‐induced constipation in rats through enhancement of fecal parameters.

Administration of loperamide was previously reported to result in the thinning of the mucus layer of the colon, which impaired the movement of contents through the colon (Shimotoyodome et al., 2000). Estimation of colonic transit time is usually adopted in evaluation of gastrointestinal disorders, including constipation. In this study, administration of loperamide also generally prolonged intestinal transit time. It has been reported that the intake of dietary fiber and polyphenol‐rich compounds causes shorter intestinal transit times, increasing water retention and causing the volume and viscosity of the intestinal contents to increase, thereby stimulating colonic mobility (Kim et al., 2016; Han et al., 2017; Spiller et al., 1980; Zhuang et al., 2019). In addition, Gorden (1989) reported that although dietary fiber has a longer transit time through the stomach and small intestine, it has a shorter transit time through the colon, resulting in a short overall time to achieve fecal excretion. According to El‐Salhy, Ystad, Mazzawi, and Gundersen (2017), high dietary fiber content might lead to more active peristalsis, resulting in the elongation of the small and large intestines. It is assumed that the length of the small and large intestines is altered to compensate for decreased nutrient absorption resulting from dietary fiber consumption. However, in this study, intestinal length was not significantly changed among experimental groups. Loperamide administration did not induce severe level of colonic inflammation since intestinal length was intact. Judd and Truswell (1985) indicated that the formation of gels by dietary fiber in the intestine might increase the weight of the intestinal contents, exert physical pressure to expand the intestinal serosa, and thereby increase the weight and length of the small intestine. It is thought that RLE rich in dietary fiber may shorten intestinal transit time, leading to the decreased number of fecal pellets in the colon. In addition, these results were similar to those of previous reports (Attaluri et al., 2011; Han et al., 2017; Kakino et al., 2010; Kim, Lee, et al., 2013; Kim, Sung, et al., 2013; Kim et al., 2018; Méité et al., 2010; Sathyanathan et al., 2013; Seo et al., 2013). Méité et al. (2010) reported that *Mareya micrantha* (Benth.) Müll. Arg. (Euphorbiaceae), which contains flavonoids, alkaloids, tannins, polyphenols, sterols, and polyterpenes, produces a significant increase in intestinal transit compared with a control group. We indicated that RLE was able to increase fecal weight and shorten intestinal transit time, and that polyphenolic compounds were responsible for those effects. Several studies have reported that phytochemicals possess laxative properties via the acetylcholine receptor in the constipated animal model (Aslam & Janbaz, 2019; Han et al., 2017; Kakino et al., 2010; Kim, Lee, et al., 2013; Kim, Sung, et al., 2013;Sathyanathan et al., 2013;Seo et al., 2013). Moreover, in a previous study, the composition of gastrointestinal microflora was improved by treatment with medicinal plants rich in polyphenolic compounds, which were effective to inhibit growth of harmful bacteria in the intestine (Lee et al., 2001). The above results suggest that RLE treatment could improve loperamide‐induced constipation in rats through stimulation of feces excretion. These laxative effects can be induced by treatment with more than 400 mg/kg of RLE. Therefore, 400 mg/kg of RLE is clinically achievable since 70 kg human may intake ~2.8 g/day. And ~2.8 g RLE consumption is ~0.35 g of fiber consumption. Considering the recommendation of daily fiber consumption, consumption of 400 mg/kg is achievable amount for clinical purpose.

Loperamide injection produces stress and is known to alter serum lipid profiles (Choi, Jeong, Cho, Cho, & Choi, 2012). Brenner, Zamecnik, Shek, and Shephard (1997) reported that serum levels of catabolic hormones including epinephrine, norepinephrine, and cortisol typically increase during periods of stress and that epinephrine may have a lipid‐mobilizing effect, since infusion of epinephrine enhances lipolysis in humans (Freyschuss, Hjemdahl, Juhlin‐Dannfelt, & Linde, 1986) and increases cholesterol content (Dimsdale, Herd, & Hartly, 1983). Therefore, many researchers measured serum lipid profiles, glucose, and ALP levels as stress factors (Kim et al., 2005). In addition, there are many enzymes, such as ALT, AST, ALP, and LDH, that are found in appreciable quantities in the serum which are not actually secreted extracellularly. It is only during tissue damage that these enzymes leak out of tissues and increase in the serum, making them marker enzymes for the safety and/or toxicity of pharmacological agents (Pendota, Yakubu, Grierson, & Afolayan, 2010). In this study, serum TG and TC levels, AI and CRF, and ALT and ALP activities were significantly lower in both RLEL and RLEH groups compared with the CON group. Moreover, many researchers have reported that total polyphenol compounds, including flavonoids in natural products, play a beneficial role in preventing cardiovascular disease as they change serum lipid levels (Giglio et al., 2018; Hertog et al., 1993). These results indicate that the stress caused by loperamide increases serum lipid levels, but RLE may improve the serum lipid profiles and prevent constipation. Improved lipid profiles may relate to the alteration of transit time. Moreover, higher fiber content in RLE may decrease biliary resorption of lipids to intestine.

Many recent studies have clearly shown the involvement of oxidative stress in constipation via elevated intracellular levels of reactive oxygen species (ROS) that damage lipids, proteins, and DNA (Rtibi et al., 2017). Loperamide enhances ROS production and induces oxidative stress (Jabri et al., 2017). Our investigation showed that injection of loperamide increased the production of MDA and H_2_O_2_ in intestinal mucosa, indicating an increase in lipid peroxidation and depletion of antioxidant enzyme activities such as SOD, CAT, and GSH‐Px. However, oral administration of RLE significantly protected against loperamide‐induced alterations in a dose‐dependent manner. The antioxidant properties of RLE are partly due to its richness in phenolic compounds, leading to higher free‐radical‐scavenging activity, while simultaneously reducing related physical changes, enhancing facile movement in the intestine. In conclusion, the present results indicate remarkable protection by RLE against loperamide‐induced alterations in oxidative stress parameters.

## 5 CONCLUSION

The results of this study demonstrate that RLE increases the total number, weight, and water content of rat fecal pellets without causing diarrhea, the incidence of which was decreased by loperamide. RLE also reduces the number of fecal pellets that remain in the colon, as well as intestinal transit time, clearly demonstrating efficacy in improving constipation. We showed that constipation is accompanied by oxidative stress, assessed by an increase in MDA level and H_2_O_2_ production, and a decrease in antioxidant enzyme activities. Treatment with RLE significantly protected against oxidative stress induced by loperamide intoxication. Phenolic compounds and dietary fiber in RLE may play key roles in the observed anticonstipation and antioxidative effects. However, further studies are required to elucidate the mechanism of the laxative effect of RLE.

## CONFLICT OF INTEREST

The authors declare no conflict of interest.

## ETHICAL APPROVAL

This study conforms to the Declaration of Helsinki, USA. All animal care and experimental protocols were ethically viewed and approved (Approval Number: CIACUC2015‐A0014) by the Institutional Animal Care and Use Committee of Chosun University, Korea.
